# Incidence of new-onset wheeze: a prospective study in a large middle-aged general population

**DOI:** 10.1186/s12890-015-0158-0

**Published:** 2015-12-16

**Authors:** Mathias Holm, Kjell Torén, Eva Andersson

**Affiliations:** Department of Occupational and Environmental Medicine, Sahlgrenska University Hospital, Box 414, SE 40530 Gothenburg, Sweden; Section of Occupational and Environmental Medicine, Institute of Medicine, Sahlgrenska Academy, University of Gothenburg, Box 414, SE 40530 Gothenburg, Sweden

**Keywords:** Wheeze, Incidence, Prospective, Predictors, Smoking, Chronic bronchitis, Rhinitis, Obesity

## Abstract

**Background:**

Wheeze is a very common respiratory symptom, which is associated with several factors and diseases. Studies on incidence of new-onset wheeze in general adult populations are rare. The present prospective study aimed to investigate the incidence rate of new-onset wheeze, and predictors for wheeze, in a general, middle-aged population.

**Methods:**

Individuals, born 1943–1973, who had participated in a previous Swedish study in 1993 (*n* = 15,813), were mailed a new respiratory questionnaire in 2003. The questionnaire, which included items about respiratory symptoms, atopy, and smoking was answered by 11,463 (72 %). Incidence rates of new-onset wheeze were calculated. Cox regression analyses were performed with incident wheeze as an event and person-years under observation as dependent variable.

**Results:**

Among those free of wheeze at baseline (*n* = 8885), there were 378 new cases of wheeze during the study period (1993–2003). The incidence rate was 4.3/1000 person-years. The adjusted risk was increased in relation to smoking (HR 2.1;95 % CI 1.7–2.7), ex-smoking (HR 1.4;95 % CI 1.1–1.9), young age (HR 1.7;95 % CI 1.3–2.2), chronic bronchitis (HR 2.3;95 % CI 0.96–5.7), and rhinitis (HR 1.8;95 % CI 1.4–2.2) at baseline, and body mass index ≥30 (HR 1.9;95 % CI 1.5–2.6) at follow-up.

**Conclusions:**

This is a unique study that presents an incidence rate for new-onset wheeze in a middle-aged, general population sample previously free of adult wheeze. The results indicate that new-onset wheeze is quite common in this age group. Health care staff should bear this in mind since new-onset wheeze could be one of the earliest symptoms of severe respiratory disease. Special attention should be paid to patients with a smoking history, chronic bronchitis, rhinitis or obesity.

## Background

Wheeze is a very common respiratory symptom caused by mechanisms narrowing airway calibre [1]. However, there are few prospective studies in general populations of adults where incidence of wheeze has been investigated and almost all of these present cumulative incidences [2–8]. Sometimes, it is not solely “wheeze” that has been investigated, but wheeze in addition to another symptom. In some studies, only current wheeze is investigated and not wheeze at any time during the study period. This probably leads to an underestimation of the “true” cumulative incidence as wheeze is a symptom that fluctuates over time. To our knowledge, there have been no prospective studies in general populations of adults presenting incidence rates of new cases of just “wheeze” using person-years.

Several factors and diseases, like asthma, chronic obstructive pulmonary disease (COPD), airway infections, obesity, tumours, and smoking have been associated with wheeze [1, 9–13]. It is a symptom that in clinical practice is often used in the diagnostic setting of different diseases. It follows that it is of great importance to be aware of conditions that can result in wheeze. In order to find predictors for a symptom or disease it is essential to conduct prospective studies.

The aim of the current prospective study was to investigate the incidence rate of new cases of wheeze in a general, middle-aged population sample, and also the relation of new-onset wheeze to smoking, age, gender, chronic bronchitis, atopy, rhinitis, and BMI.

## Methods

In 1993, we sent 20,000 individuals randomly selected from a county in western Sweden a short questionnaire which comprised items about respiratory symptoms and smoking [14]. They were all born between 1943 and 1973. Those answering the questionnaire were mailed a follow-up questionnaire in 2003 and the response rate was 72 % (11,463 out of 15,813) [15]. After exclusion of four subjects with unreasonable stop smoking data and 52 individuals that could not be linked to the 1993 questionnaire, 11,407 remained. To get a baseline population free of wheeze, subjects who at baseline reported adult (>15 years old) wheeze (*n* = 2005), or who at follow-up reported year of onset before 1993 (*n* = 334, 74 % >15 years old at onset) or reported wheeze but no year of onset (*n* = 88) were excluded from the analyses. Subjects reporting asthma but not wheeze (*n* = 95) were also excluded. An affirmative answer to “Have you been diagnosed as having asthma by a physician?” and/or “Do you have, or have you ever had, asthma?” defined asthma [16]. In total, 8885 subjects were included in the study. In the 1993 survey, there were 12,402 subjects who reported that they were free of adult asthma and wheeze. The follow-up questionnaire contained new questions as well as questions identical to those in the baseline questionnaire. The study has been approved by the local Committee of Ethics in Gothenburg (Ö 192–03). Informed consent was obtained from all study participants.

Wheeze was defined as a positive answer to the following question: “Have you, since the age of 15, ever had whistling or wheezing in your chest?” It was followed by a question: “How old were you when these symptoms started?” The same questions were used in an incident study of wheeze among bleachery workers performed by our research group [17]. To be defined as having chronic bronchitis, individuals had to answer positively to all three of the following questions: “Have you, since the age of 15, suffered from long-standing cough with sputum?,” “If yes, has any period lasted for at least 3 months?,” and “If yes, have you had such periods for at least 2 consecutive years?” This is in line with the internationally accepted diagnostic criterion for chronic bronchitis, of a chronic productive cough for at least 3 months a year for 2 consecutive years [18, 19].

Individuals were classified as having atopy if they gave a positive answer to “Do you have, or have you ever had, hay fever?” and/or “Do you have, or have you ever had, atopic dermatitis?” Rhinitis was defined by a positive answer to “Have you, since the age of 15, ever had nasal symptoms other than hay fever, like nasal blockage and/or sneezing without having a cold?” [20]. Smoking status was checked in both 1993 and 2003 and year of smoke-start and smoke-stop was asked for. Body mass index (BMI) was calculated from reported weight and height in the 2003 questionnaire using the formula BMI = mass (kg)/(height (m))^2^.

For most of the analyses, the SAS statistical package, version 9.2 (SAS Institute, Cary, NC, USA), was used. The incidence analyses were conducted using STATA (College Station, TX, USA). Based on reported year of onset, the incidence rate of wheeze (number of new cases/1000 person-years) with 95 % confidence intervals (CIs) was calculated for the study period (1993–2003). Subjects contributed person-years until onset of wheeze. Person-years were broken down into non-smoking, smoking, and ex-smoking years, respectively. Ex-smokers contributed smoking years until 2 years after smoking cessation. Incidence rate ratios were calculated, comparing smoking person-years with non-smoking person-years and ex-smoking person-years with non-smoking person-years, respectively. In the Cox regression analyses (PROC PHREG), incident wheeze was the event of interest and person-years under observation the dependent variable. The models were performed for all years as well as for smoking, ex-smoking, and non-smoking years, respectively. The explanatory variables used were gender, atopy, chronic bronchitis, rhinitis, BMI ≥30, and different age groups (20–30 years, 31–40 years, 41–50 years), which were simultaneously included. Current smoking and ex-smoking were included when applying a model with all subjects. Hazard ratios (HRs) are given with 95 % CIs. In order to study loss to follow-up, the 8885 individuals included in the study were compared with the entire population free of asthma and adult wheeze at baseline (*n* = 12,402) using methods described by Johannessen et al. (2014) [21].

## Results

The 8885 subjects who were included in the study represented a total of 86,955 person-years. The demographic characteristics of these subjects are shown in Table 1. In 1993, the smoking prevalence was around 25 %. By 2003, it had fallen to 15 %.

**Table 1:** Demographic characteristics of the study population at baseline in 1993, by smoking group

	Smokers	Ex-smokers	Never-smokers	All^a^
Age, years (SD)	37.0 (8.8)	39.9 (7.5)	34.3 (9.1)	36.1 (9.0)
All	24.8 %^b^ (*n* = 2202)	20 %^b^ (*n* = 1777)	50.8 %^b^ (*n* = 4509)	100 % (*n* = 8885)
Female gender	58.1 % (*n* = 1279)	49.6 % (*n* = 881)	51.2 % (*n* = 2308)	52.5 % (*n* = 4665)
Atopy	13.1 % (*n* = 288)	16.9 % (*n* = 301)	18.5 % (*n* = 836)	16.8 % (*n* = 1490)
Rhinitis	23.3 % (*n* = 513)	27.7 % (*n* = 493)	22.6 % (*n* = 1021)	23.9 % (*n* = 2121)
Chronic bronchitis	0.6 % (*n* = 13)	0.7 % (*n* = 12)	0.6 % (*n* = 25)	0.6 % (*n* = 52)
BMI ≥30 (2003)	10.2 % (*n* = 224)	11.4 % (*n* = 202)	8.8 % (*n* = 395)	9.7 % (*n* = 858)
20-30 years old	27.4 % (*n* = 603)	13.8 % (*n* = 246)	40.3 % (*n* = 1815)	31.8 % (*n* = 2829)
31-40 years old	32.2 % (*n* = 709)	32.4 % (*n* = 575)	29.6 % (*n* = 1335)	30.7 % (*n* = 2723)
41-50 years old	40.4 % (*n* = 890)	53.8 % (*n* = 956)	30.1 % (*n* = 1359)	37.5 % (*n* = 3333)

*SD* standard deviation, *BMI* body mass index

^a^Includes also those with missing smoking data

^b^Percent of all individuals in the study

During the study period (1993–2003), there were 378 new cases of wheeze. The crude incidence rate was 4.3/1000 person-years and the cumulative incidence was around 4 %. Table 2 gives incidence rates and relative risk of wheeze for women, men, atopics, non-atopics, individuals with or without chronic bronchitis, subjects with or without rhinitis, participants with BMI ≥30 or <30, and different age groups in relation to smoking. Smoking seems to increase the risk for incident wheeze in all groups (cases among subjects with chronic bronchitis were too few to be analysed) (Table 2).

**Table 2:** New-onset adult wheeze in relation to gender, atopy, rhinitis, CB, age, and BMI

	All years, 1993-2003	Smoking years	Ex-smoking years	Never-smoking years	Incidence rate ratios (smoking/never- smoking years)	Incidence rate ratios (ex- /never- smoking years)
All	4.3 (3.9–4.8)	7.9 (6.8–9.3)	4.2 (3.0–4.7)	3.0 (2.5–3.6)	2.6 (2.1–3.3)	1.3 (0.9–1.7)
Women	4.8 (4.2–5.5)	8.3 (6.8–10.1)	4.8 (3.6–6.3)	2.9 (2.3–3.7)	2.8 (2.1–3.9)	1.6 (1.1–2.4)
Men	3.8 (3.3–4.5)	7.4 (5.6–9.5)	2.9 (2.0–4.1)	3.1 (2.4–3.9)	2.4 (1.6–3.4)	1.4 (0.96–2.1)
Atopic	4.9 (3.8–6.2)	8.2 (5.2–12.5)	5.3 (3.2–8.3)	3.6 (2.4–5.1)	2.3 (1.3–4.2)	1.5 (0.8–2.7)
Non-atopic	4.2 (3.7–4.7)	7.9 (6.6–9.4)	3.5 (2.7–4.5)	2.8 (2.3–3.4)	2.8 (2.1–3.7)	1.3 (0.9–1.7)
Rhinitis	6.6 (5.5–7.8)	11.4 (8.5–14.9)	5.5 (3.8–7.8)	5.0 (3.7–6.6)	2.3 (1.5–3.4)	1.1 (0.7–1.8)
Non-rhinitis	3.6 (3.2–4.1)	7.0 (5.7–8.5)	3.1 (2.3–4.1)	2.5 (2.0–3.1)	2.8 (2.1–3.8)	1.2 (0.8–1.8)
CB *(5 cases)*	10.0 (3.3–23)	10.1 (0.2–56)	6.2 (0.1–35)	13.4 (2.8–39)	---	---
Non-CB	4.3 (3.9–4.7)	7.9 (6.7–9.3)	3.8 (3.0–4.7)	3.0 (2.5–3.5)	2.7 (2.1–3.4)	1.3 (0.9–1.7)
BMI ≥30 (2003)	7.6 (5.9–9.8)	14.3 (9.3–21)	6.1 (3.4–10.1)	5.5 (3.4–8.5)	2.6 (1.4–4.8)	1.1 (0.5–2.2)
BMI < 30 (2003)	4.0 (3.5–4.4)	7.3 (6.1–8.6)	3.5 (2.7–4.5)	2.8 (2.3–3.3)	2.6 (2.0–3.4)	1.3 (0.9–1.7)
20–30 years old	5.2 (4.3–6.1)	10.0 (7.6–12.9)	4.8 (2.9–7.6)	3.7 (2.9–4.7)	2.7 (1.9–3.9)	1.3 (0.7–2.2)
31–40 years old	3.6 (2.9–4.4)	6.4 (4.6–8.7)	3.2 (2.0–7.6)	2.5 (1.7–3.6)	2.5 (1.6–4.1)	1.3 (0.7–2.2)
41–50 years old	4.2 (3.5–5.0)	7.7 (5.9–9.8)	3.8 (2.8–5.2)	2.6 (1.8–3.6)	3.0 (1.9–4.7)	1.5 (0.9–2.4)

The data are presented as incidence rates (cases/1000 person-years) and relative risks, by smoking exposure (95 % confidence intervals). Atopy, rhinitis, CB, and age at baseline in 1993, and BMI in 2003, respectively

*CB* chronic bronchitis, *BMI* body mass index

Table 3 presents the results of Cox regression. The Table shows an increased relative risk for wheeze among those who were current smokers or ex-smokers at baseline, compared to those who were never-smokers. Young age and having chronic bronchitis were both risk factors for wheeze, but the increased risk of incident wheeze among those with chronic bronchitis was only significant among never-smokers, probably due to small numbers of subjects (Table 3). Those reporting rhinitis at baseline also had an increased risk of wheeze. On the other hand, atopy adjusted for rhinitis did not seem to increase the risk for wheeze. Among those with rhinitis, atopy was significantly more common compared to those who were free of rhinitis (28.2 % vs. 13.3 %). Finally, obesity at follow-up in 2003 was associated with incident wheeze (Table 3).

**Table 3:** Hazard ratios based on Cox regression analysis of adult new-onset wheeze, by smoking exposure

	Smoking years	Ex-smoking years	Never-smoking years	All
Current smoking at baseline^b^	NA^a^	NA^a^	NA^a^	2.1 (1.7–2.7)
Ex-smoking at baseline^b^	NA^a^	NA^a^	NA^a^	1.4 (1.1–1.9)
Female gender^b^	1.1 (0.8–1.5)	1.6 (0.99–2.4)	0.9 (0.7–1.3)	1.1 (0.9–1.4)
Rhinitis at baseline^b^	1.7 (1.2–2.4)	1.6 (1.0–2.6)	2.0 (1.4–2.9)	1.8 (1.4–2.2)
Atopic at baseline^b^	0.9 (0.5–1.4)	1.3 (0.8–2.3)	1.1 (0.7–1.6)	1.0 (0.8–1.3)
CB at baseline^b^	1.2 (0.2–8.9)	1.7 (0.2–12.6)	4.5 (1.4–14)	2.3 (0.96–5.7)
20–30 years at baseline^b^	1.7 (1.1–2.5)	1.8 (0.9–3.4)	1.5 (1.0–2.4)	1.7 (1.3–2.2)
41–50 years at baseline^b^	1.2 (0.8–1.8)	1.5 (0.8–2.5)	1.1 (0.6–1.7)	1.2 (0.9–1.6)
BMI ≥30 (2003)^b^	2.1 (1.4–3.2)	1.9 (1.1–3.4)	2.0 (1.2–3.2)	1.9 (1.5–2.6)

The data are presented as hazard ratios (95 % confidence intervals), based on Cox regression analysis. One Cox regression model per column. Atopy, rhinitis, CB, and age at baseline in 1993, and BMI in 2003, respectively

*CB* chronic bronchitis, *BMI* body mass index

^a^NA = not applicable

^b^1 = yes, and 0 = no

The 8885 individuals that responded to the 2003 questionnaire were compared with the entire population free of asthma and adult wheeze at baseline (*n* = 12,402). Response to the 2003 questionnaire was associated with female sex, older age and non-smoking. However, baseline prevalence of atopy, rhinitis and chronic bronchitis did not differ when comparing responders to the 2003 questionnaire with all baseline participants free of asthma and adult wheeze. The associations of age, sex, and smoking habits with the respiratory outcomes chronic bronchitis and rhinitis at baseline did not differ significantly when comparing the two populations.

## Discussion

This prospective study in a middle-aged, general population showed an incidence rate of 4.3/1000 person-years for new-onset adult wheeze, giving a cumulative incidence of approximately 4 % during the study period. Smoking doubled the risk of incident wheeze and ex-smoking was associated with a 40 % increased risk. Young age, chronic bronchitis, and rhinitis were also predictors for incident wheeze. In addition, obesity at follow-up was associated with an increased risk of incident wheeze.

To our knowledge, the current study is the first to report an incidence rate of new-onset wheeze based on person-years in an adult, general population sample previously free of adult wheeze. An observed incidence rate is dependent on the baseline population and it has been shown that the composition of this population is of great importance when estimating asthma incidence in prospective studies, and probably the same will be true when incidence of wheeze is studied [22]. An accurate incidence rate also relies on correct reporting of previous symptoms [23]. In our study, we excluded all subjects with previous self-reported wheeze since the age of 15 and/or who had ever had asthma, which is essential for a correct incidence rate of new-onset adult wheeze. Even the characterization of the outcome is significant. Few prospective studies have investigated incidence of wheeze in general adult populations [2–8] and sometimes it is not just “wheeze” that has been investigated, but wheeze in addition to another symptom or an asthma diagnosis that has been equated with wheeze. Most of the studies only present cumulative incidence. In an approximately 30-year, follow-up Scottish study of subjects aged 39–45 years and free of childhood wheezing, 11.5 % of 1542 reported adult onset wheeze [5]. This is similar to our results given that our data is adjusted to a longer study period. A British cohort study of individuals born during 1 specific week in 1958 reported a cumulative incidence for wheeze without asthma of 10.5 % when those free of wheeze and asthma were followed between the ages of 34 and 42 years [7]. It is difficult to compare these results with the results from our study since the British study investigated individuals born during a specific week and we studied a general population with a wide age range. Hedlund et al*.* report a 10-year cumulative incidence of wheeze of 10 % in a population-based study from northern Sweden [6]. The divergent results, compared to our study, could perhaps be explained by different exclusion criteria at baseline where Hedlund et al*.* seem to have excluded only those with present wheeze. In a general population study from our research group, 3.3 % of 1506 subjects reported new-onset wheeze during a 4 year follow-up [8], which is a higher incidence than in the current study. In the present study, subjects were followed for a longer period of time and some individuals with minor and transient wheeze early during the study period may have forgotten their symptoms. However, analysing year of onset of wheeze did not support this hypothesis because new cases were relatively evenly distributed over the study period (data not shown). Dodge and Burrows present high incidence rates for new-onset “attacks of shortness of breath with wheeze” in a random sample of non-Mexican,-white American households in Tucson, Arizona, USA, followed for approximately 3.5 years [2]. More than 10 % of their population developed “attacks of shortness of breath with wheeze” during the study period. It appears that the subjects could have entered the incident study even if they had reported wheeze without shortness of breath at baseline. This could, of course, have contributed to the high incidence rate found.

Apart from the interest in an accurate incidence rate of new-onset wheeze we also wanted to study predictors of wheeze. Smoking has been reported to be associated with wheeze, especially in women [13]. Our prospective study revealed smoking as a strong predictor for wheeze in both sexes. Smoking doubled the risk of wheeze and subjects who had stopped smoking had an increased risk of around 40 % for incident wheeze. A positive trend was that the smoking prevalence among the participants of our study had fallen from 25 % in 1993 to 15 % in 2003, which certainly affects the total incidence rate of wheeze.

In the current study, few subjects with chronic bronchitis were included. Many individuals with chronic bronchitis in 1993 were excluded from the present study as they also reported asthma and/or wheeze. Nevertheless, chronic bronchitis at baseline was a significant risk factor for future wheeze in never-smoking subjects. Chronic bronchitis is dependent on bronchial hyper-secretion, and has been closely related to COPD, which may explain the association with wheeze [12].

We found that a high BMI at follow-up was associated with incident wheeze, which is in line with a cross-sectional study where an association was found between severe obesity and wheeze [10]. Interestingly, the authors of that study did not find any increased airway responsiveness or airway obstruction in the obese group compared with a normal BMI group. In a report from the American Thoracic Society, it was concluded that obesity and asthma are closely associated, but that obesity only has a small impact on airway hyper responsiveness and airway obstruction (ratio of forced expiratory volume in 1 s to the forced vital capacity of the lungs (FEV_1_/FVC ratio)) [24]. It therefore seems that obesity is related to respiratory symptoms like wheeze, but also to an asthma diagnosis.

In our study, the youngest age group (20–30 years at baseline) had a significantly increased risk for incident wheeze. This is in contrast to previous studies where incidence of wheeze increased with age [2, 4, 6]. However, compared to our cohort, the mean age in two of these study populations was approximately 12 years and 16 years higher, respectively, which makes comparisons difficult [4, 6]. We could also see a trend for an increased incidence of wheeze in subjects aged 41–50 years at baseline compared with subjects aged 31–40 years, which is in line with these previous findings.

Rhinitis, but not atopy defined as ever had hay fever or atopic dermatitis, at baseline was associated with new-onset wheeze in the current study. Among those with rhinitis, there were more than twice as many with atopy compared to those free of rhinitis. Atopy is related to asthma and often, allergic rhinitis precedes the asthma diagnosis [25, 26]. However, even non-allergic rhinitis is a potent predictor of adult-onset asthma [25, 26]. In our study, all individuals with wheeze at baseline, including those with atopy, were excluded. This means that subjects with ever hay fever or atopic dermatitis, even during childhood, who had developed wheeze or asthma, were excluded. The remaining individuals with atopy therefore represented a select group and it seems that adults with atopy are not at risk of developing new-onset wheeze. However, the increased risk for wheeze in those with rhinitis may reflect asthma not yet diagnosed [26].

Socio-economic status and environmental factors other than smoking have been associated with wheeze [5, 6, 27]. It would have been very interesting to study these presumptive predictors of new-onset wheeze, but we do not have baseline information about these factors.

The prospective design of the present study allows the identification of predictors of incident wheeze, and it also reduces recall bias. The high response rate among the large number of subjects invited to participate increases the potential for the study population to represent the target population. However, selection bias is a problem in epidemiological studies. In the literature, studies comparing prevalence of respiratory symptoms and airway diseases between responders and non-responders, respectively, diverge in their findings [28–30], and it has been suggested that all studies should include analysis of non-responders [31]. In our 1993 survey, non-response was associated with lower age, male sex, being foreign-born, being unmarried, unemployment, and low income [14]. Response to the 2003 questionnaire was related to older age, female sex and non-smoking. However, it seems that exclusion of those lost to follow-up did not alter the prevalence of respiratory symptoms or exposure-outcome associations at baseline. Despite some limitations of the current study, there are good reasons to believe that the results of the study could be valid for other, similar populations.

## Conclusions

The present prospective study is unique as it presents an incidence rate for new-onset wheeze in a middle-aged, general population previously free of adult wheeze. The results indicate that new-onset wheeze is quite common in middle-aged people. It is thus important for health care staff to be aware of this fact since new-onset wheeze could be the first sign of severe respiratory diseases, like COPD, asthma, airway infections, and tumours. Special attention should be paid to patients with a smoking history, chronic bronchitis, rhinitis or obesity.
